# Photon exchange and entanglement formation during transmission through a rectangular quantum barrier

**DOI:** 10.1016/j.physleta.2015.05.010

**Published:** 2015-09-04

**Authors:** Georg Sulyok, Katharina Durstberger-Rennhofer, Johann Summhammer

**Affiliations:** Institute of Atomic and Subatomic Physics, Vienna University of Technology, 1020 Vienna, Austria

**Keywords:** Quantum mechanics, Quantum optics, Scattering, Rectangular potential, Particle–field-entanglement, Photon exchange

## Abstract

When a quantum particle traverses a rectangular potential created by a quantum field both photon exchange and entanglement between particle and field take place. We present the full analytic solution of the Schrödinger equation of the composite particle–field system allowing investigation of these phenomena in detail and comparison to the results of a classical field treatment. Besides entanglement formation, remarkable differences also appear with respect to the symmetry between energy emission and absorption, resonance effects and if the field initially occupies the vacuum state.

## Highlights

• Full analytic solution for the transmission through a rectangular quantum barrier.
• Transition from classical to quantum field is illustrated.
• Entanglement as a unique feature of the quantized field treatment is emphasized.
• Non-trivial entanglement between position and photon number for coherent fields.
• Interaction with vacuum state peculiar to the quantized field treatment.

## 1 Introduction

The behavior of a quantum particle exposed to an oscillating rectangular potential has been studied by several authors under different aspects involving, for example, tunneling time [1,2], chaotic signatures [3,4], appearance of Fano resonances [5], Floquet scattering for strong fields [6] and its absence for non-Hermitian potentials [7], chiral tunneling [8], charge pumping [9] and other photon assisted quantum transport phenomena in theory [10–12] and experiment [13–18], recently realized particularly in quantum dots [19–22].

In these works, though the potential is treated as a classical quantity, the change of the particle's energy is explicitly attributed to a photon emission or absorption process. Here, we introduce the photon concept in a formally correct way by describing the field generating the potential as quantized. Hence, we pursue the ideas which we started to elaborate in our previous publication [23]. There, we only arrived at an algebraic expression for the photon transition amplitudes whereas we now are able to present analytic results for all important initial field states enabling advanced investigations on photon exchange processes and entanglement formation.

In order to compare semiclassical and fully-quantized treatment in our physical scenario, we recapitulate the results of the calculation for a classical field (Section 2). Then, we turn to the quantized field treatment (Section 3). After presenting the general algebraic solution, we will explicitly evaluate the photon exchange probabilities for an incoming plane wave and for a field being initially in an arbitrary Fock state, a thermal state or a coherent state. The special cases of no initial photons (vacuum state) and of high initial photon numbers will be treated in particular.

## 2 Classical treatment of the field

The potential created by a classical field is a real-valued function of space and time in the particle's Hamiltonian. Our considered potential oscillates harmonically in time and is spatially constant for 0≤x<L and vanishes outside.

(1) $$\overset{ˆ}{H}=\left\{ \begin{array}{cc} \frac{{\overset{ˆ}{p}}^{2}}{2m}+V\cos(\omega t+\varphi ), & \text{if }0\leq x<L\text{ (region II)}\\ \frac{{\overset{ˆ}{p}}^{2}}{2m}, & \text{else (region }I+\mathrm{III}\text{)} \end{array}\right.$$

**Fig. 1:**
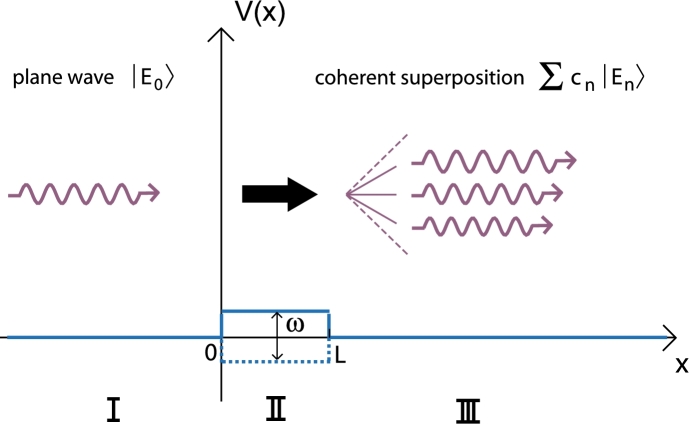
(Color online.) Spatial characteristics of the considered potential *V*. It is harmonically oscillating in time with frequency *ω* in region II and vanishes elsewhere. Behind the barrier the incoming plane wave is split up into a coherent superposition of plane waves with energy $E_{n}=E_{0}+n\hbar \omega$.

It therefore corresponds to a harmonically oscillating rectangular potential barrier (see Fig. 1).

The Schrödinger equation is solved in each of the three regions separately and then the wave functions are matched by continuity conditions. A general approach based on Floquet theory [24] can be found in [25]. Since the calculation of transmission and reflection coefficients requires the solution of an infinite dimensional equation system no closed analytic expression for them is feasible (see Chapter II of [25]). However, we can deduce from Chapter III of [25] that the transmission probability approaches one if the incoming energy $E_{0}$ becomes large with respect to the potential amplitude *V* and the associated “photon” energy *ħω* ($E_{0}\gg V,\hbar \omega$). For the further, we restrict ourselves to incoming waves whose energy $E_{0}$ is sufficiently high so that reflection at the barrier can be neglected. In that case, standard methods for differential equations suffice to find the solution [26,27]. If we assume the wave function $|\psi_{I}\rangle$ in region I to be a plane wave with wave vector $k_{0}$ we get for the wave function $|\psi_{\text{III}}\rangle$ behind the potential barrier

(2) $$|\psi_{I}\rangle =|k_{0}\rangle \;⟹\;|\psi_{\text{III}}\rangle =\sum_{n=-\infty }^{+\infty }J_{n}(\beta )\;e^{-in\eta }\;|k_{n}\rangle$$

where

(3) $$\beta =2\frac{V}{\hbar \omega }\sin\frac{\omega \tau }{2},\;\eta =\varphi +\frac{\omega \tau }{2}+\frac{\pi }{2}$$

(4) $$\tau =\frac{mL}{\hbar k_{0}}=\frac{L}{v_{0}},\;k_{n}^{2}=k_{0}^{2}+\frac{2m}{\hbar }n\omega$$

For a more detailed derivation including the solution for region II as well we refer to [26,28].

In summary, a plane wave $|k_{0}\rangle$ gets split up into a coherent superposition of plane waves $|k_{n}\rangle$ whose energy is given by the incident energy $E_{0}$ plus integer multiples of *ħω*. The transition probability for an energy exchange of *nħω* is just the square of the Bessel function $J_{n}^{\;2}$ of the *n*-th order. The argument of the Bessel function shows that an increasing amplitude *V* of the potential also increases the probability for exchanging larger amounts of energy.

Apart from this expected result, it also exhibits a geometric “resonance”-condition. If the “time-of-flight” *τ* through the field region and the oscillation frequency are tuned such that ωτ=2lπ, l∈N, all Bessel functions $J_{n}$ with n≠0 vanish and no energy is transferred at all. The plane wave even passes the potential completely unaltered since $J_{0}(0)=1$. That's a remarkable difference between an oscillating and a static potential where at least phase factors are always attached to the wave function. An experimental implementation of the classical potential can be found in [28,29].

## 3 Quantized treatment of the field

Since the energy exchange between the harmonically oscillating potential and the particle is quantized by integer multiples of *ħω* most authors already speak of photon exchange processes although the potential stems from a purely classical field. This notion is problematic since a formally correct introduction of the photon concept requires a quantization of the field generating the potential. For this purpose, the corresponding field equation has to be solved and a canonical quantization condition for Fourier amplitudes of the field is introduced which are then no longer complex-valued coefficients but interpreted as creation and annihilation operators.

For the further, we assume that such a quantum field whose spatial mode is well approximated by the rectangular form generates the potential. The quantum system we observe now consists of particle and field together. The total state |Ψ〉 of the composite quantum system is an element of the product Hilbert space $H_{\text{total}}=H_{\text{particle}}⊗H_{\text{field}}$. If the particle is outside the field region the evolution of the state is given by ${\overset{ˆ}{H}}_{0}$ composed of the free single-system Hamiltonians ${\overset{ˆ}{h}}_{0}^{p}$ and ${\overset{ˆ}{h}}_{0}^{f}$ of particle and field

(5) $${\overset{ˆ}{H}}_{0}={\overset{ˆ}{h}}_{0}^{p}⊗1+1⊗{\overset{ˆ}{h}}_{0}^{f}$$

(6) $${\overset{ˆ}{h}}_{0}^{p}=\frac{{\overset{ˆ}{p}}^{2}}{2m},\;{\overset{ˆ}{h}}_{0}^{f}=\hbar \omega \left({\overset{ˆ}{a}}^{†}\overset{ˆ}{a}+\frac{1}{2}\right)$$

**Fig. 2:**
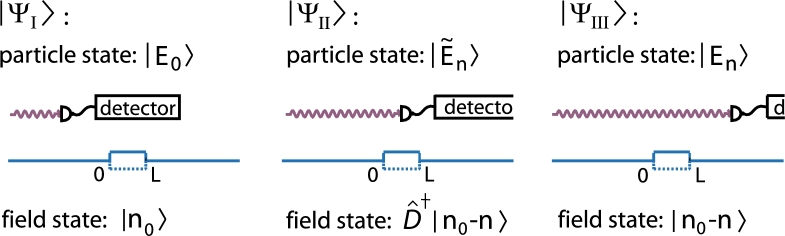
(Color online.) In the quantized field treatment, the particle's position determines which of the overall wavefunctions $|\Psi_{I}\rangle$, $|\Psi_{\text{II}}\rangle$ or $|\Psi_{\text{III}}\rangle$ describes the state of the composite quantum system. The spatial characteristics of the field do not change, it is always present between 0 and L, but the field state changes in accordance with the particle due to their interaction.

Interaction between field and particle takes place if the particle is inside the field region which can be formally expressed by using the Heavyside *θ*-function in the quantized version of the sinusoidal driving term

(7) $${\overset{ˆ}{H}}_{\text{int}}=\lambda \;(\theta (\overset{ˆ}{x})-\theta (\overset{ˆ}{x}-L))⊗\left({\overset{ˆ}{a}}^{†}+\overset{ˆ}{a}\right)$$

where all constants have already been absorbed in the coupling parameter *λ*. Since the sheer presence of an interaction is connected to the particle's position we again distinguish between three different states $|\Psi_{I}\rangle$, $|\Psi_{\text{II}}\rangle$, and $|\Psi_{\text{III}}\rangle$ for the composite quantum system (see Fig. 2).

### 3.1 Fock states

As in the classical field case, we assume that the kinetic energy of the incoming particle is sufficiently high so that reflection at field entry can be neglected. Then, we can choose as ansatz for $|\Psi_{I}\rangle$ the particle's state to be a single plane wave with wave vector $k_{0}$ and the field to be present in a distinct Fock state $n_{0}$

(8) $$|\Psi_{I}\rangle =|k_{0}\rangle ⊗|n_{0}\rangle$$

In order to get $|\Psi_{\text{II}}\rangle$, we switch to the position space representation of the particle's part of the wave function and match $|\Psi_{I}\rangle$ at $x_{\text{particle}}\equiv x=0$ for all times *t* with the general solution in region II. It is given by an arbitrary linear superposition of plane waves for the particle and displaced Fock states for the field [23]. The continuity conditions uniquely determine the expansion coefficients and yet $|\Psi_{\text{II}}\rangle$. At x=L, $|\Psi_{\text{II}}\rangle$ has to be matched with the general solution of the free Hamiltonian which is given by an arbitrary superposition of plane waves and Fock states. The state $|\Psi_{\text{III}}\rangle$ behind the field region then reads

(9) $$|\Psi_{\text{III}}\rangle =\sum_{n=0}^{\infty }t_{n_{0}n}|k_{n_{0}-n}\rangle ⊗|n\rangle ,\;k_{l}^{2}=k_{0}^{2}+\frac{2m}{\hbar }l\omega$$

with

(10) $$t_{n_{0}n}=e^{i{\overset{¯}{\lambda }}^{2}\omega \tau }\sum_{q=0}^{\infty }\langle n|{\overset{ˆ}{D}}^{†}(\overset{¯}{\lambda })|q\rangle \langle q|\overset{ˆ}{D}(\overset{¯}{\lambda })|n_{0}\rangle e^{-i(q-n)\omega \tau }$$

where $\overset{ˆ}{D}$ denotes the displacement operator, $\overset{¯}{\lambda }=\lambda /\hbar \omega$ the coupling constant in units of the photon energy, and $\tau =mL/\hbar k_{0}$ the “time of flight” through the field region as in the classical case (Eq. (4)). Details of the calculation as well as the explicit result for $|\Psi_{\text{II}}\rangle$ can be found in [23]. The matrix $t_{n_{0}n}$ gives the amplitudes for the transition from an initial photon number $n_{0}$ to the final photon number *n*. The wave vector of the traversing particle changes accordingly from $k_{0}$ to $k_{n_{0}-n}$. Every emission of field quanta is absorbed in the kinetic energy of the particle and vice versa. The final state is the coherent superposition of all such combinations $|k_{n_{0}-n}\rangle$ and |n〉 and therefore highly entangled.

The algebraic form of the transition matrix $t_{n_{0}n}$ (Eq. (10)) can be further developed in order to get an analytic expression. The calculation is straightforward, but rather lengthy and requires the nontrivial Kummer transformation formula for confluent hypergeometric functions. The key steps are outlined in Appendix A. Finally we arrive at

(11) $$t_{n_{0}n}=e^{i\Phi }\sqrt{\frac{n_{0}!}{n!}}\;e^{-\frac{\Lambda^{2}}{2}}\;\Lambda^{n-n_{0}}\;L_{n_{0}}^{n-n_{0}}(\Lambda^{2})$$

where $L_{n}^{\alpha }(x)$ denotes the generalized Laguerre polynomial and

(12) $$\Phi ={\overset{¯}{\lambda }}^{2}\left(\omega \tau -\sin\omega \tau \right)+(n-n_{0})(\frac{\omega \tau }{2}-\frac{\pi }{2})$$

(13) $$\Lambda =2\overset{¯}{\lambda }\sin\frac{\omega \tau }{2}$$

The coupling strength parameter Λ indicates the capacity of the particle–field system to exchange energy and contains the coupling constant *λ* (in units of *ħω*) and the sinusoidal resonance factor that already occurred the classical treatment. The probability that the initial photon number $n_{0}$ changes to the final photon number *n* after the transmission of the particle through the field is given by $P_{n_{0},n}={\left|t_{n_{0}n}\right|}^{2}$.

(14) $$P_{n_{0},n}=\frac{n_{0}!}{n!}\;e^{-\Lambda^{2}}\;{\left(\Lambda^{2}\right)}^{n-n_{0}}\;{\left(L_{n_{0}}^{n-n_{0}}(\Lambda^{2})\right)}^{2}$$

**Fig. 3:**
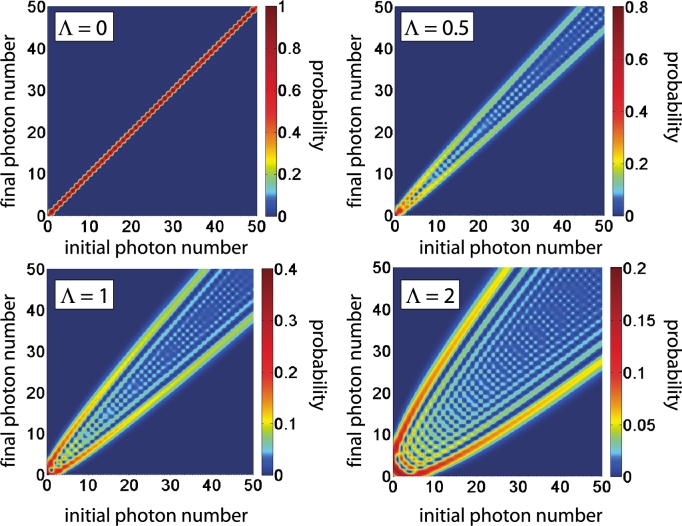
(Color online.) Transition probabilities $P_{n_{0},n}$ for initial photon numbers $n_{0}$ (plotted on the abscissa) and final photon number *n* (indicated on the ordinata) for coupling strengths $\Lambda =2\frac{\lambda }{\hbar \omega }\sin\frac{\omega \tau }{2}=0.0$, Λ = 0.5, Λ = 1.0, Λ = 2.0.

In Fig. 3, the transition probabilities $P_{n_{0},n}$ for various coupling strengths Λ are depicted. As in the classical case, the probability for exchanging higher number of photons increases with increasing coupling strength, but absorption and emission of the same number of photons are not equally probable. We have in general $P_{n_{0},n}=P_{n,n_{0}}$ but $P_{n_{0},n_{0}+q}\neq P_{n_{0},n_{0}-q}$. This asymmetry is reflected in the expectation values of the energy of particle and field after the interaction process.

(15) $$\langle \Psi_{\text{III}}|{\overset{ˆ}{h}}_{0}^{p}⊗1|\Psi_{\text{III}}\rangle =\frac{\hbar^{2}k_{0}^{2}}{2m}-\hbar \omega \Lambda^{2}$$

(16) $$\langle \Psi_{\text{III}}|1⊗h_{0}^{f}|\Psi_{\text{III}}\rangle =\hbar \omega \left(n_{0}+\Lambda^{2}+\frac{1}{2}\right)$$

Since we assumed a high energetic incoming particle for which reflection could be neglected the net energy transfer goes from particle to field. Not until the initial photon number becomes large with respect to the normed coupling constant $n_{0}\gg \overset{¯}{\lambda }$ the symmetry between emission and absorption is restored. We can then use from the appendix of [30]

(17) $$\langle n_{0}+l|\overset{ˆ}{D}(\overset{¯}{\lambda })|n_{0}+r\rangle =J_{l-r}(2\overset{¯}{\lambda }\sqrt{n_{0}}),\;n_{0}\gg \overset{¯}{\lambda }$$

and apply Graf's addition theorem for Bessel functions in (Eq. (10)) to get

(18) $$P_{n_{0},n_{0}+q}=J_{q}{\left(2\Lambda \sqrt{n_{0}}\right)}^{2}=P_{n_{0},n_{0}-q},\;n_{0}\gg \overset{¯}{\lambda }$$

Large initial photon numbers indicate the transition to the classical field regime, and indeed, the Bessel function in (Eq. (18)) is reminiscent of the classical result (Eq. (2)). But, if we trace over the field state the particle is still present in an incoherent superposition of the $|k_{n}\rangle$ weighted with the $J_{n}^{2}$ as to be expected from the entangled total state $|\Psi_{\text{III}}\rangle$. A proper transition from the quantum to the classical case can only be achieved by starting with a coherent field state (see Section 3.4).

If the length *L* of the field region and the wave vector $k_{0}$ are tuned such that the “resonance” condition ωτ=2πn, n∈N is fulfilled no energy between particle and field is transferred as in the classical case. But, contrary to the classical treatment, an overall phase factor remains in form of $|\Psi_{\text{III}}\rangle =e^{i{\overset{¯}{\lambda }}^{2}\omega \tau }|k_{0}\rangle ⊗|n_{0}\rangle$ and could be accessible in an interferometric setup.

### 3.2 Vacuum state

Another remarkable feature of the quantum field treatment can be revealed from the investigation of the vacuum state. For a classical field, vacuum is realized by simply setting the potential to zero resulting in an unaltered, free evolution of the particle's plane wave ($|\psi_{I}\rangle =|\psi_{\text{III}}\rangle =|k_{0}\rangle$). In the quantized treatment, vacuum is represented by an initial Fock state $|n_{0}=0\rangle$ which still interacts with the particle and yields as final state $|\Psi_{\text{III}}\rangle$ behind the field region

(19) $$|\Psi_{I}\rangle =|k_{0}\rangle ⊗|0\rangle \;\Rightarrow \;|\Psi_{\text{III}}\rangle =\sum_{n=0}^{\infty }t_{0n}|k_{-n}\rangle ⊗|n\rangle$$

with a photon exchange probability

(20) $$P_{0,n}={\left|t_{0n}\right|}^{2}=\frac{1}{n!}\;e^{-\Lambda^{2}}\;\Lambda^{2n}$$

The particle thus transfers energy to the vacuum field leading to a Poissonian distributed final photon number. Let's consider, for example, a superconducting resonant circuit as source of the field. The magnetic field along the axis of a properly shaped coil is well approximated by the rectangular form. A particle with a magnetic dipole moment passing through the coil then interacts with the circuit and excites it with a measurable loss of kinetic energy even if the circuit is initially uncharged and there is classically no field it can couple to. The phenomenon that vacuum in quantum field theory does not mean to “no influence” as known from Casimir forces or Lamb shift is clearly visible here as well.

### 3.3 Thermal state

In realistic experimental situations, the pure vacuum state can not be achieved. Due to unavoidable coupling to the environment acting as heat bath with a finite temperature *T* higher photon numbers are excited as well and we encounter an incoherent, so-called thermal state $\rho_{\text{thermal}}$ for the field

(21) $$\rho_{\text{thermal}}=\sum_{n=0}^{\infty }y^{n}(1-y)|n\rangle \langle n|,\;y=e^{-\frac{\hbar \omega }{k_{B}T}}$$

**Fig. 4:**
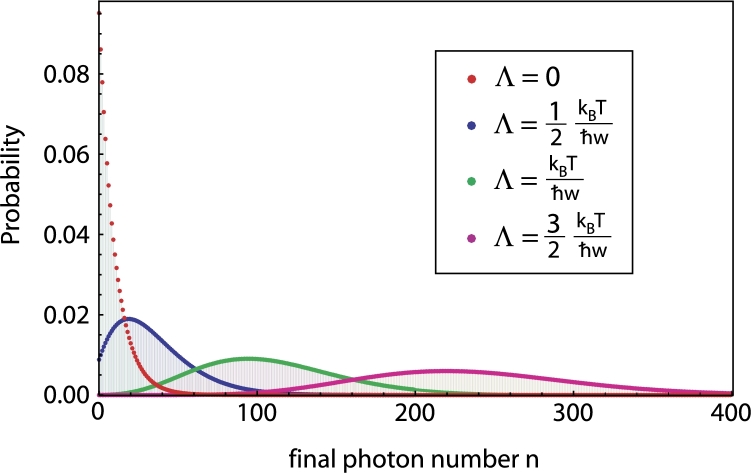
(Color online.) Probability distribution of the final photon number for different coupling strengths $\Lambda =2\frac{\lambda }{\hbar \omega }\sin\frac{\omega \tau }{2}$ if the field was initially in a thermal state (temperature *T*, $k_{B}T/\hbar \omega \approx 10$).

We now choose the field to be initially in such a thermal state. After the particle has traversed the field region, the probability $P_{n}^{\text{therm}}$ of finding the field in a distinct Fock state |n〉 is given by

(22) $$P_{n}^{\text{therm}}=e^{-\Lambda^{2}(1-y)}\;(1-y)\;y^{n}L_{n}(-\frac{\Lambda^{2}{\left(1-y\right)}^{2}}{y})$$

where $L_{n}$ denotes the ordinary Laguerre polynomial. As depicted in Fig. 4, the initial thermal distribution changes as soon as the coupling strength Λ reaches the order of $k_{B}T/\hbar \omega$.

### 3.4 Coherent state

Now, we consider the field to be initially in a coherent state |α〉 labeled by the complex number $\alpha =|\alpha |e^{i\varphi_{\alpha }}$

(23) $$|\Psi_{I}\rangle =|k_{0}\rangle ⊗|\alpha \rangle ,\;|\alpha \rangle =e^{-\frac{{\left|\alpha \right|}^{2}}{2}}\sum_{n=0}^{\infty }\frac{\alpha^{n}}{\sqrt{n!}}|n\rangle$$

For the further evaluation of this expression we start from the algebraic form of the transition matrix (Eq. (10)) and work in the position representation of the particle's part of wave function. Expansion of the wave vectors $k_{n}$ (Eq. (9)) around the initial wave vector $k_{0}$ enables us to absorb phase factors in the coherent state and evaluate the displacements. The projection onto the position eigenstate $|x\rangle \in H_{\text{particle}}$ after the transmission reads

(24) $$\langle x|\Psi_{\text{III}}\rangle =e^{i{\overset{¯}{\lambda }}^{2}\omega \tau }e^{-i{\overset{¯}{\lambda }}^{2}\sin\omega \tau }e^{ik_{0}x}e^{i\Lambda |\alpha |\sin(\varphi_{\Lambda }(x)-\varphi_{\alpha })}|\alpha +\Lambda e^{i\varphi_{\Lambda }(x)}\rangle$$

where

(25) $$\varphi_{\Lambda }(x)=\frac{\omega \tau }{2}-\frac{\omega }{v_{0}}x-\frac{\pi }{2}$$

**Fig. 5:**
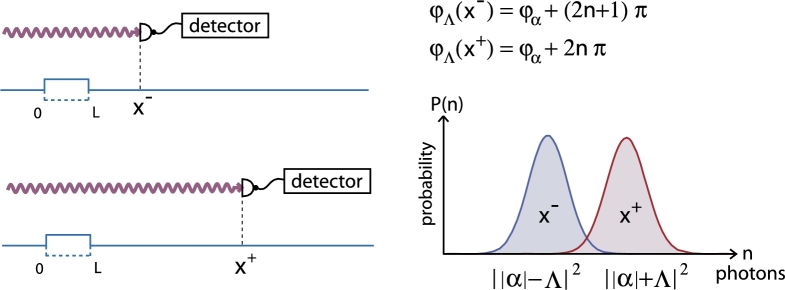
(Color online.) Through the phase $\varphi_{\Lambda }(x)$, the final coherent state $|\alpha +\Lambda e^{i\varphi_{\Lambda }(x)}\rangle$ depends on the particle's position. Detecting high (low) photon numbers in the field is therefore correlated to positions $x^{+}$ ($x^{-}$) and vice versa.

The entanglement between particle and field is now indicated by the explicit occurrence of the particle's position coordinate *x* in the final (coherent) field state. If the particle is detected at a certain position $x_{1}$ the field state is projected onto $|\alpha +\Lambda e^{i\varphi_{\Lambda }(x_{1})}\rangle$. We can now place two detectors at positions $x^{+}$ and $x^{-}$ which satisfy

(26) $$\varphi_{\Lambda }(x^{+})\equiv \varphi_{\Lambda }^{+}=\varphi_{\alpha }+2n\pi$$

(27) $$\varphi_{\Lambda }(x^{-})\equiv \varphi_{\Lambda }^{-}=\varphi_{\alpha }+2(m-1)\pi$$

where *n* and *m* are arbitrary integers and take a look at the photon number distributions of the related coherent states. The phases $\varphi_{\Lambda }$ are chosen such that the average photon numbers are given by $||\alpha {\left|+\Lambda \right|}^{2}$ for $x^{+}$ and $||\alpha {\left|-\Lambda \right|}^{2}$ for $x^{-}$ respectively. For a sufficiently high coupling strength $\Lambda ≳\frac{1}{2}$ the corresponding distributions cease to overlap. Detecting the particle around $x^{-}$ thus increases the probability of having roughly $||\alpha {\left|-\Lambda \right|}^{2}$ photons in the field whereas detection around $x^{+}$ is connected to an average photon number of $||\alpha {\left|+\Lambda \right|}^{2}$. Likewise, finding $||\alpha {\left|+\Lambda \right|}^{2}$ photons in the field determines the particle's position to be around $x^{+}$ and analogously for $x^{-}$ (see Fig. 5). The photon number thus contains information about the particle's position.

**Fig. 6:**
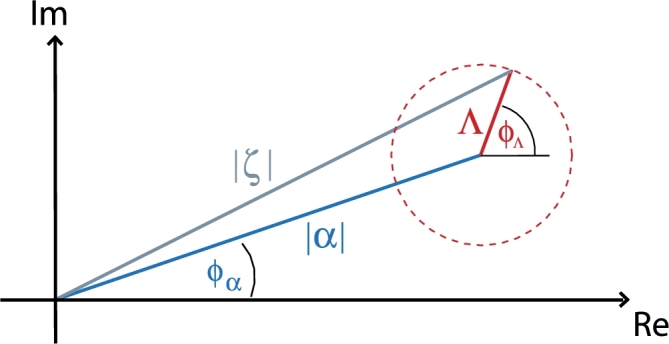
(Color online.) For a coherent initial field state |*α*〉 the field state after the transmission is given by an incoherent mixture ∫*dx* |*ξ*〉〈*ξ*| of all coherent states $|\xi \rangle =|\alpha +\Lambda e^{i\varphi_{\Lambda }}\rangle$.

If no measurement on the particle is carried out the field state is obtained from the total density matrix $\rho =|\Psi_{\text{III}}\rangle \langle \Psi_{\text{III}}|$ by performing the partial trace over the particle's degrees of freedom. We get an incoherent mixture of coherent states for the field's density matrix

(28) $$\rho_{\text{field}}=\int dx|\alpha +\Lambda e^{i\varphi_{\Lambda }(x)}\rangle \langle \alpha +\Lambda e^{i\varphi_{\Lambda }(x)}|$$

which can be illustrated in the Fresnel plane (see Fig. 6).

Like in case of Fock states, on average, the particle transfers energy to the field as indicated by the expectation values

(29) $$\langle \Psi_{\text{III}}|{\overset{ˆ}{h}}_{0}^{p}⊗1|\Psi_{\text{III}}\rangle =\frac{\hbar^{2}k_{0}}{2m}-\hbar \omega \Lambda^{2}$$

(30) $$\langle \Psi_{\text{III}}|1⊗{\overset{ˆ}{h}}_{0}^{p}|\Psi_{\text{III}}\rangle =\hbar \omega \left({\left|\alpha \right|}^{2}+\Lambda^{2}+\frac{1}{2}\right)$$

If we increase the mean photon number such that we can neglect the coupling strength Λ against |α| we can simplify (Eq. (24)) and arrive at

(31) $$|\Psi_{\text{III}}\rangle =e^{i{\overset{¯}{\lambda }}^{2}\omega \tau }e^{-i{\overset{¯}{\lambda }}^{2}\sin\omega \tau }\sum_{n=-\infty }^{+\infty }J_{n}(\Lambda |\alpha |)e^{-in\eta }|k_{n}\rangle ⊗|\alpha \rangle$$

where we have use the abbreviation *η* of the classical section (Eq. (3)) with $\varphi_{\alpha }\;\overset{ˆ}{=}-\varphi$. Disregarding the back action of the particle on the field thus leads to a simple product state of the composite quantum system and therefore to disentanglement. By tracing over the field, we obtain the particle's state which is now a coherent superposition of $|k_{n}\rangle$ weighted with the Bessel functions $J_{n}$ and a phase factor $e^{-in\eta }$ as in the classical case. A general survey on the correspondence between time-independent Schrödinger equations for the composite particle–field system and time-dependent Schrödinger equations for the particle alone that contain the expression for the classical field as potential term can be found in [31].

If we choose the initial coherent state |α〉 to be the vacuum state |0〉 and therefore set α=0 in Eq. (24) we consistently end up with the same final state as in Eq. (19).

At resonance (ωτ=2πn, n∈N), no photon exchange takes place and the initial state again only obtains an overall phase factor and becomes $|\Psi_{\text{III}}\rangle =e^{i{\overset{¯}{\lambda }}^{2}\omega \tau }|k_{0}\rangle ⊗|\alpha \rangle$ after the interaction.

## 4 Conclusion

The quantum mechanical scattering on a rectangular potential created by a quantum field is completely analytically solvable for incoming particles whose energy is high enough to neglect reflections. Transition amplitudes and photon exchange probabilities can be entirely expressed in terms of standard functions for the most important types of initial field states, that is, Fock, thermal, and coherent states.

The quantized treatment of both particle and field reveals their entanglement in the interaction process. Therefore, the setup could be of interest for quantum information applications where a spatially fixed (field) and a moveable component (particle) are required. For Fock states, entanglement actually occurs between the energy eigenstates of the particle and the photon number states of the field, but, for a coherent initial field state, the particle's position and the photon number get entangled.

Since the Hamiltonian treated here represents the fully quantized version of the Tien–Gordon model which successfully describes transport in ac-driven nanostructures and forms the basis of all photon-assisted transport phenomena experimental tests of our predictions are feasible once the quantum state of the oscillating device is known. Superconducting resonant circuits whose quantum features have been studied extensively in circuit qed could also provide the quantum barriers discussed here.

The Schrödinger equation of the composite system is time-independent and thus, the total energy is conserved in the transmission process. Though, photon emission and absorption are in general not equally probable, on average, the high-energetic, incoming particle transfers energy to the field. Only if the photon number in the field becomes large, the symmetry between emission and absorption is restored. However, for high number Fock states, entanglement is nevertheless maintained and the energy transfer happens incoherently. Just for coherent field states whose mean photon number is so high against the coupling strength that the influence of the particle on the field can be neglected the transition to the classical, coherent energy exchange becomes visible.

A remarkable feature of the fully quantized treatment is the interaction with the vacuum. Though from the classical point of view a free evolution of the particle should take place, the particle transfers energy to the field and their combined state changes.

For the experimentally more realistic situation of not a pure vacuum but a thermal field state visible effects occur once the coupling constant becomes comparable to the thermal energy ($k_{B}T$) of the environmental heat bath.

At resonance, that is when the length of the field region and the particle's wavelength are related such that destructive interference suppresses any photon exchange, the wave function nevertheless changes and obtains an overall phase factor. In the quantized treatment, a completely unaltered evolution only happens in the trivial case of a vanishing coupling constant.

The investigation of the rectangular quantum potential already exhibits the formation of entanglement between particle and field as it is to be expected for arbitrarily shaped potentials as well, though with modified transition probabilities. Extension to 2- or 3-dimensional scattering potentials could give rise to entanglement between the field state and the spatial direction of the outgoing wave thus also deployable in entanglement based quantum information applications.

## Appendix A Derivation of the analytic expression of the transition amplitude

In order to derive an analytic expression for the matrix $t_{n_{0}n}$ yielding the probability amplitudes for the transition from an initial photon number $n_{0}$ to the final photon number *n* we start with its algebraic form as given by (Eq. (10))

(A.1) $$t_{n_{0}n}=e^{i{\overset{¯}{\lambda }}^{2}\omega \tau }\sum_{q=0}^{\infty }\langle n|{\overset{ˆ}{D}}^{†}(\overset{¯}{\lambda })|q\rangle \langle q|\overset{ˆ}{D}(\overset{¯}{\lambda })|n_{0}\rangle e^{-i(q-n)\omega \tau }$$

By using standard identities of the Fock states, the creation/annihilation operators $\overset{ˆ}{a}/{\overset{ˆ}{a}}^{†}$ and the displacement operator $\overset{ˆ}{D}$ we rewrite

(A.2) $$\langle n|{\overset{ˆ}{D}}^{†}(\overset{¯}{\lambda })|q\rangle =\langle 0|\frac{{\overset{ˆ}{a}}^{n}}{\sqrt{n!}}\;{\overset{ˆ}{D}}^{†}(\overset{¯}{\lambda })|q\rangle =\frac{1}{\sqrt{n!}}\langle \overset{¯}{\lambda }|{\left(\overset{ˆ}{a}-\overset{¯}{\lambda }\right)}^{n}|q\rangle$$

Note, that $\langle \overset{¯}{\lambda }|$ stands for a coherent state and not a Fock state. We now exploit the binomial theorem and apply the creation operators (with exponents *k*) to the Fock states |q〉 which are then projected onto the coherent state $\langle \overset{¯}{\lambda }|$ yielding its (q−k)-th expansion coefficient. We proceed analogously with the matrix element $\langle q|\overset{ˆ}{D}(\overset{¯}{\lambda })|n_{0}\rangle$ and are thus left with analytic summands only. The summations can be further performed if we exploit the nontrivial identity

(A.3) $$\sum_{q=0}^{\infty }\frac{(q+k)!}{(q+k-l)!}\frac{x^{q}}{q!}=\sum_{q=0}^{\infty }\left(\begin{array}{c} l\\ q \end{array}\right)\left(\begin{array}{c} k\\ q \end{array}\right)\;q!\;x^{l-q}\;e^{x}$$

which can be traced back to the Kummer transformation formula for confluent hypergeometric functions. This can be seen most easily when the sums from (A.3) are written as Kummer's function M(a,b,z) (left sum) and as Tricomi's function U(c,d,y) respectively (right sum).

After we have used identity (A.3) on the summation over *q* the two other sums can be evaluated with the help of the binomial theorem. The remaining summation over *q* can be conveniently expressed with the generalized Laguerre polynomial $L_{n}^{\alpha }(x)$ which leads to the following compact analytic result for the transition matrix $t_{n_{0}n}$

(A.4) $$t_{n_{0}n}=e^{i\Phi }\sqrt{\frac{n_{0}!}{n!}}\;e^{-\frac{\Lambda^{2}}{2}}\;\Lambda^{n-n_{0}}\;L_{n_{0}}^{n-n_{0}}(\Lambda^{2})$$

where

(A.5) $$\Phi ={\overset{¯}{\lambda }}^{2}\left(\omega \tau -\sin\omega \tau \right)+(n-n_{0})(\frac{\omega \tau }{2}-\frac{\pi }{2})$$

(A.6) $$\Lambda =2\overset{¯}{\lambda }\sin\frac{\omega \tau }{2}.$$
